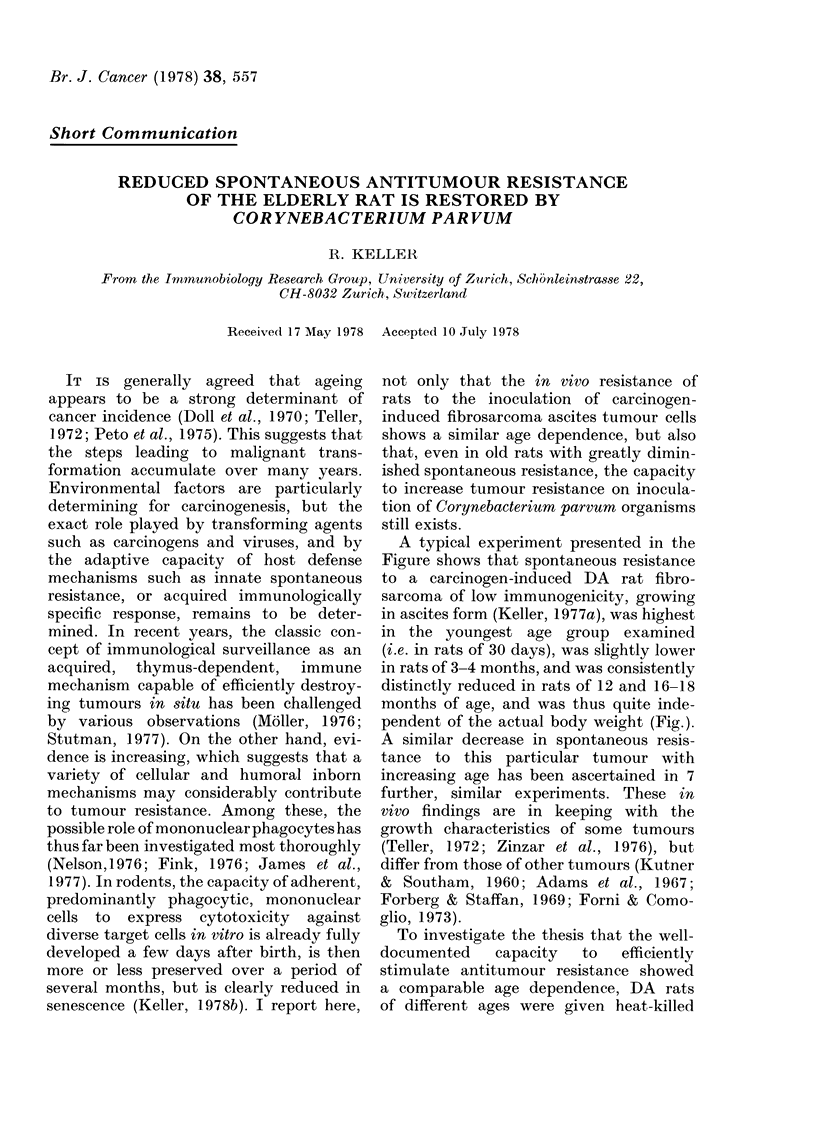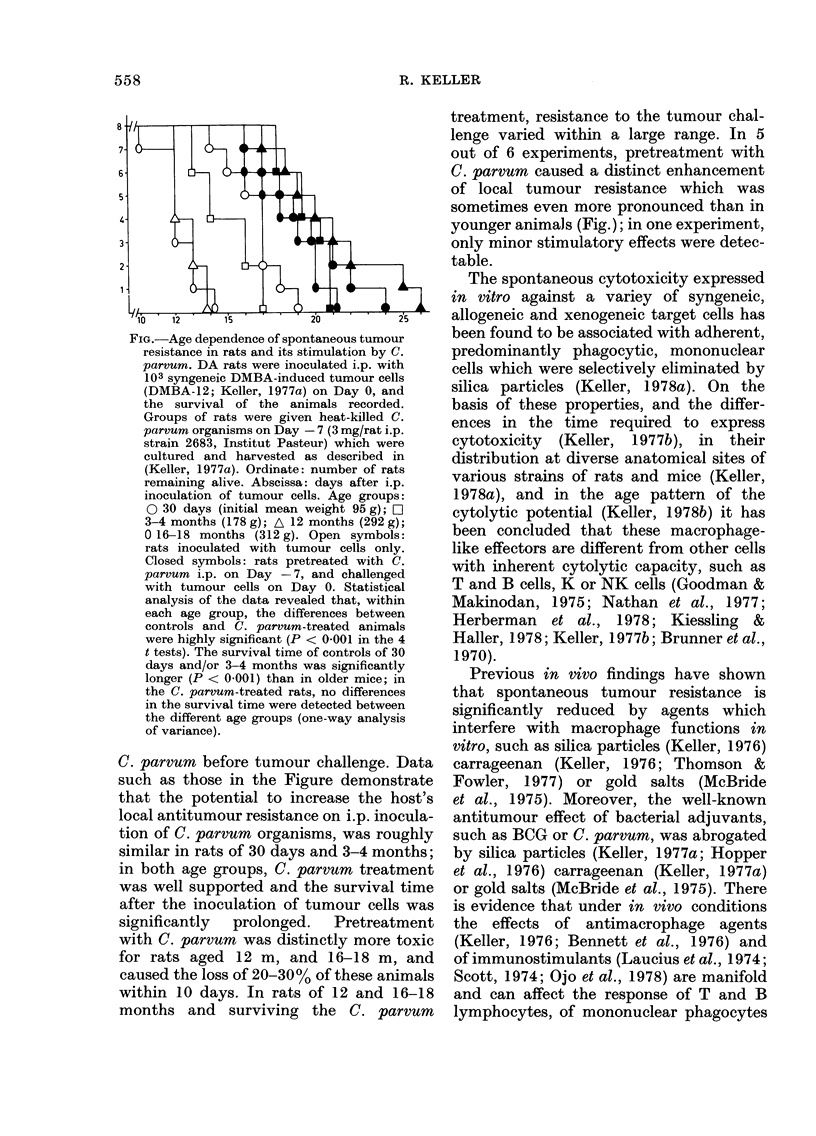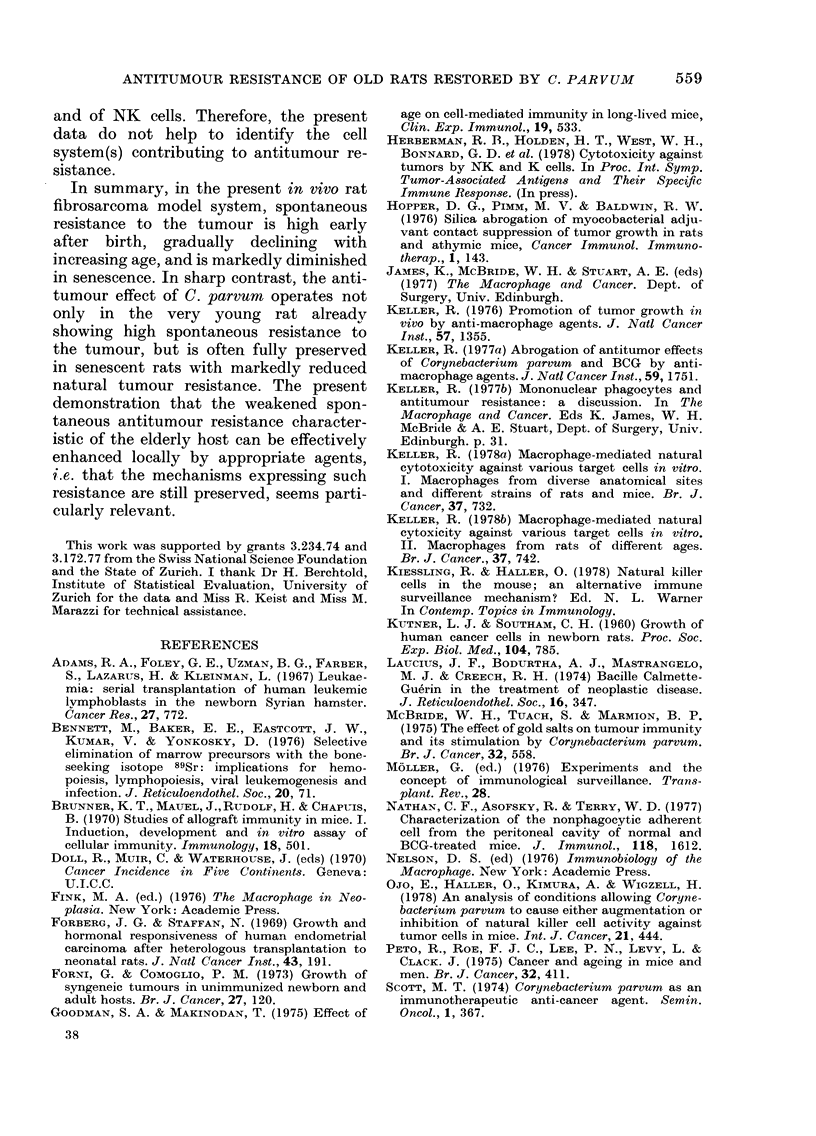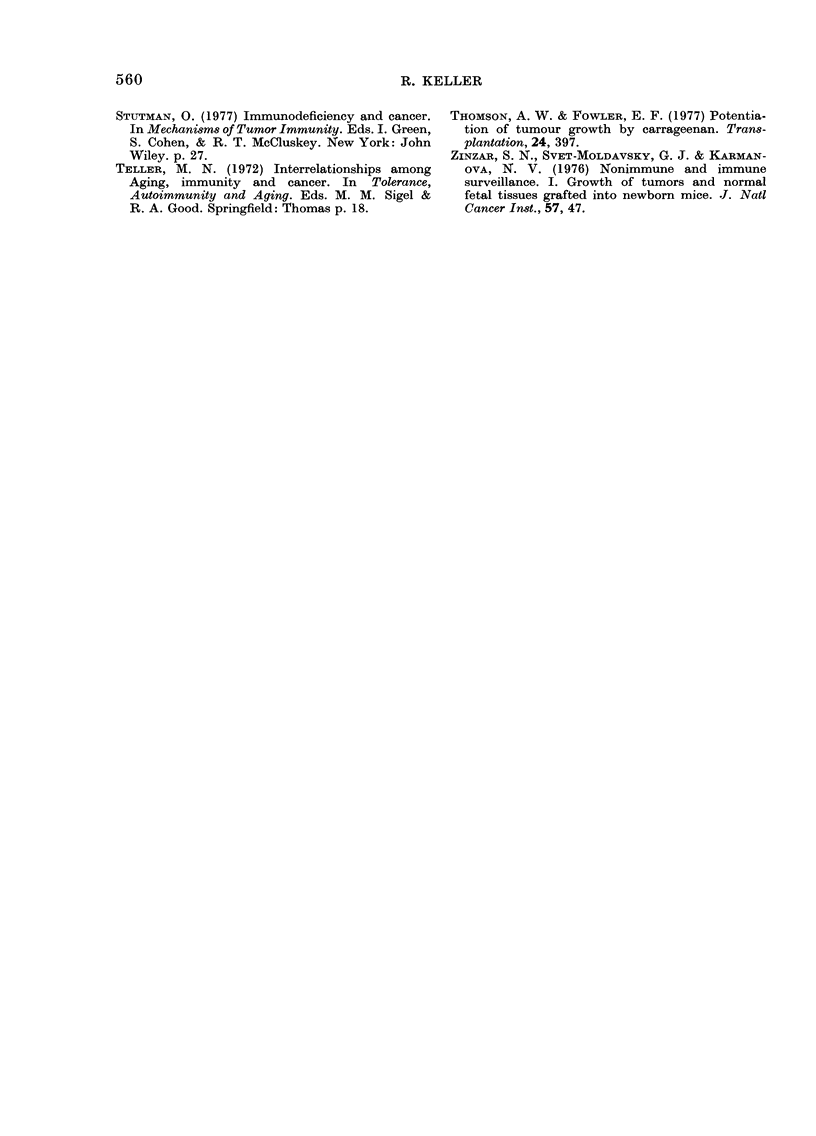# Reduced spontaneous antitumour resistance of the elderly rat is restored by Corynebacterium parvum.

**DOI:** 10.1038/bjc.1978.245

**Published:** 1978-10

**Authors:** R. Keller


*Br. J. Cancer* (1978) **38**, 557

***Short Communication***

### REDUCED SPONTANEOUS ANTITUMOUR RESISTANCE OF THE ELDERLY RAT IS RESTORED BY *CORYNEBACTERIUM PARVUM*

R. KELLER

*From the Immunobiology Research Group, University of Zurich, Schönleinstrasse 22, CH-8032 Zurich, Switzerland*

Received 17 May 1978 Accepted 10 July 1978

It is generally agreed that ageing appears to be a strong determinant of cancer incidence (Doll *et al.*, 1970; Teller, 1972; Peto *et al.*, 1975). This suggests that the steps leading to malignant transformation accumulate over many years. Environmental factors are particularly determining for carcinogenesis, but the exact role played by transforming agents such as carcinogens and viruses, and by the adaptive capacity of host defense mechanisms such as innate spontaneous resistance, or acquired immunologically specific response, remains to be determined. In recent years, the classic concept of immunological surveillance as an acquired, thymus-dependent, immune mechanism capable of efficiently destroying tumours *in situ* has been challenged by various observations (Möller, 1976; Stutman, 1977). On the other hand, evidence is increasing, which suggests that a variety of cellular and humoral inborn mechanisms may considerably contribute to tumour resistance. Among these, the possible role of mononuclear phagocytes has thus far been investigated most thoroughly (Nelson, 1976; Fink, 1976; James *et al.*, 1977). In rodents, the capacity of adherent, predominantly phagocytic, mononuclear cells to express cytotoxicity against diverse target cells *in vitro* is already fully developed a few days after birth, is then more or less preserved over a period of several months, but is clearly reduced in senescence (Keller, 1978*b*). I report here, not only that the *in vivo* resistance of rats to the inoculation of carcinogen-induced fibrosarcoma ascites tumour cells shows a similar age dependence, but also that, even in old rats with greatly diminished spontaneous resistance, the capacity to increase tumour resistance on inoculation of *Corynebacterium parvum* organisms still exists.

A typical experiment presented in the Figure shows that spontaneous resistance to a carcinogen-induced DA rat fibrosarcoma of low immunogenicity, growing in ascites form (Keller, 1977*a*), was highest in the youngest age group examined (*i.e.* in rats of 30 days), was slightly lower in rats of 3–4 months, and was consistently distinctly reduced in rats of 12 and 16–18 months of age, and was thus quite independent of the actual body weight (Fig.). A similar decrease in spontaneous resistance to this particular tumour with increasing age has been ascertained in 7 further, similar experiments. These *in vivo* findings are in keeping with the growth characteristics of some tumours (Teller, 1972; Zinzar *et al.*, 1976), but differ from those of other tumours (Kutner & Southam, 1960; Adams *et al.*, 1967; Forberg & Staffan, 1969; Forni & Comoglio, 1973).

To investigate the thesis that the well-documented capacity to efficiently stimulate antitumour resistance showed a comparable age dependence, DA rats of different ages were given heat-killed

FIG.—Age dependence of spontaneous tumour resistance in rats and its stimulation by *C. parvum*. DA rats were inoculated i.p. with $10^3$ syngeneic DMBA-induced tumour cells (DMBA-12; Keller, 1977*a*) on Day 0, and the survival of the animals recorded. Groups of rats were given heat-killed *C. parvum* organisms on Day $-7$ (3 mg/rat i.p. strain 2683, Institut Pasteur) which were cultured and harvested as described in (Keller, 1977*a*). Ordinate: number of rats remaining alive. Abscissa: days after i.p. inoculation of tumour cells. Age groups: ○ 30 days (initial mean weight 95 g); □ 3–4 months (178 g); △ 12 months (292 g); 0 16–18 months (312 g). Open symbols: rats inoculated with tumour cells only. Closed symbols: rats pretreated with *C. parvum* i.p. on Day $-7$, and challenged with tumour cells on Day 0. Statistical analysis of the data revealed that, within each age group, the differences between controls and *C. parvum*-treated animals were highly significant ($P < 0{\cdot}001$ in the 4 *t* tests). The survival time of controls of 30 days and/or 3–4 months was significantly longer ($P < 0{\cdot}001$) than in older mice; in the *C. parvum*-treated rats, no differences in the survival time were detected between the different age groups (one-way analysis of variance).

*C. parvum* before tumour challenge. Data such as those in the Figure demonstrate that the potential to increase the host's local antitumour resistance on i.p. inoculation of *C. parvum* organisms, was roughly similar in rats of 30 days and 3–4 months; in both age groups, *C. parvum* treatment was well supported and the survival time after the inoculation of tumour cells was significantly prolonged. Pretreatment with *C. parvum* was distinctly more toxic for rats aged 12 m, and 16–18 m, and caused the loss of 20–30% of these animals within 10 days. In rats of 12 and 16–18 months and surviving the *C. parvum* treatment, resistance to the tumour challenge varied within a large range. In 5 out of 6 experiments, pretreatment with *C. parvum* caused a distinct enhancement of local tumour resistance which was sometimes even more pronounced than in younger animals (Fig.); in one experiment, only minor stimulatory effects were detectable.

The spontaneous cytotoxicity expressed *in vitro* against a variey of syngeneic, allogeneic and xenogeneic target cells has been found to be associated with adherent, predominantly phagocytic, mononuclear cells which were selectively eliminated by silica particles (Keller, 1978*a*). On the basis of these properties, and the differences in the time required to express cytotoxicity (Keller, 1977*b*), in their distribution at diverse anatomical sites of various strains of rats and mice (Keller, 1978*a*), and in the age pattern of the cytolytic potential (Keller, 1978*b*) it has been concluded that these macrophage-like effectors are different from other cells with inherent cytolytic capacity, such as T and B cells, K or NK cells (Goodman & Makinodan, 1975; Nathan *et al.*, 1977; Herberman *et al.*, 1978; Kiessling & Haller, 1978; Keller, 1977*b*; Brunner *et al.*, 1970).

Previous *in vivo* findings have shown that spontaneous tumour resistance is significantly reduced by agents which interfere with macrophage functions *in vitro*, such as silica particles (Keller, 1976) carrageenan (Keller, 1976; Thomson & Fowler, 1977) or gold salts (McBride *et al.*, 1975). Moreover, the well-known antitumour effect of bacterial adjuvants, such as BCG or *C. parvum*, was abrogated by silica particles (Keller, 1977*a*; Hopper *et al.*, 1976) carrageenan (Keller, 1977*a*) or gold salts (McBride *et al.*, 1975). There is evidence that under *in vivo* conditions the effects of antimacrophage agents (Keller, 1976; Bennett *et al.*, 1976) and of immunostimulants (Laucius *et al.*, 1974; Scott, 1974; Ojo *et al.*, 1978) are manifold and can affect the response of T and B lymphocytes, of mononuclear phagocytes and of NK cells. Therefore, the present data do not help to identify the cell system(s) contributing to antitumour resistance.

In summary, in the present *in vivo* rat fibrosarcoma model system, spontaneous resistance to the tumour is high early after birth, gradually declining with increasing age, and is markedly diminished in senescence. In sharp contrast, the antitumour effect of *C. parvum* operates not only in the very young rat already showing high spontaneous resistance to the tumour, but is often fully preserved in senescent rats with markedly reduced natural tumour resistance. The present demonstration that the weakened spontaneous antitumour resistance characteristic of the elderly host can be effectively enhanced locally by appropriate agents, *i.e.* that the mechanisms expressing such resistance are still preserved, seems particularly relevant.

This work was supported by grants 3.234.74 and 3.172.77 from the Swiss National Science Foundation and the State of Zurich. I thank Dr H. Berchtold, Institute of Statistical Evaluation, University of Zurich for the data and Miss R. Keist and Miss M. Marazzi for technical assistance.

### REFERENCES

ADAMS, R. A., FOLEY, G. E., UZMAN, B. G., FARBER, S., LAZARUS, H. & KLEINMAN, L. (1967) Leukaemia: serial transplantation of human leukemic lymphoblasts in the newborn Syrian hamster. *Cancer Res.*, **27**, 772.

BENNETT, M., BAKER, E. E., EASTCOTT, J. W., KUMAR, V. & YONKOSKY, D. (1976) Selective elimination of marrow precursors with the bone-seeking isotope $^{89}$Sr: implications for hemopoiesis, lymphopoiesis, viral leukemogenesis and infection. *J. Reticuloendothel. Soc.*, **20**, 71.

BRUNNER, K. T., MAUEL, J., RUDOLF, H. & CHAPUIS, B. (1970) Studies of allograft immunity in mice. I. Induction, development and *in vitro* assay of cellular immunity. *Immunology*, **18**, 501.

DOLL, R., MUIR, C. & WATERHOUSE, J. (eds) (1970) *Cancer Incidence in Five Continents.* Geneva: U.I.C.C.

FINK, M. A. (ed.) (1976) *The Macrophage in Neoplasia.* New York: Academic Press.

FORBERG, J. G. & STAFFAN, N. (1969) Growth and hormonal responsiveness of human endometrial carcinoma after heterologous transplantation to neonatal rats. *J. Natl Cancer Inst.*, **43**, 191.

FORNI, G. & COMOGLIO, P. M. (1973) Growth of syngeneic tumours in unimmunized newborn and adult hosts. *Br. J. Cancer*, **27**, 120.

GOODMAN, S. A. & MAKINODAN, T. (1975) Effect of age on cell-mediated immunity in long-lived mice, *Clin. Exp. Immunol.*, **19**, 533.

HERBERMAN, R. B., HOLDEN, H. T., WEST, W. H., BONNARD, G. D. *et al.* (1978) Cytotoxicity against tumors by NK and K cells. In *Proc. Int. Symp. Tumor-Associated Antigens and Their Specific Immune Response.* (In press).

HOPPER, D. G., PIMM, M. V. & BALDWIN, R. W. (1976) Silica abrogation of myocobacterial adjuvant contact suppression of tumor growth in rats and athymic mice, *Cancer Immunol. Immunotherap.*, **1**, 143.

JAMES, K., MCBRIDE, W. H. & STUART, A. E. (eds) (1977) *The Macrophage and Cancer.* Dept. of Surgery, Univ. Edinburgh.

KELLER, R. (1976) Promotion of tumor growth *in vivo* by anti-macrophage agents. *J. Natl Cancer Inst.*, **57**, 1355.

KELLER, R. (1977*a*) Abrogation of antitumor effects of *Corynebacterium parvum* and BCG by anti-macrophage agents. *J. Natl Cancer Inst.*, **59**, 1751.

KELLER, R. (1977*b*) Mononuclear phagocytes and antitumour resistance: a discussion. In *The Macrophage and Cancer.* Eds K. James, W. H. McBride & A. E. Stuart, Dept. of Surgery, Univ. Edinburgh. p. 31.

KELLER, R. (1978*a*) Macrophage-mediated natural cytotoxicity against various target cells *in vitro.* I. Macrophages from diverse anatomical sites and different strains of rats and mice. *Br. J. Cancer*, **37**, 732.

KELLER, R. (1978*b*) Macrophage-mediated natural cytoxicity against various target cells *in vitro.* II. Macrophages from rats of different ages. *Br. J. Cancer.*, **37**, 742.

KIESSLING, R. & HALLER, O. (1978) Natural killer cells in the mouse; an alternative immune surveillance mechanism? Ed. N. L. Warner In *Contemp. Topics in Immunology.*

KUTNER, L. J. & SOUTHAM, C. H. (1960) Growth of human cancer cells in newborn rats. *Proc. Soc. Exp. Biol. Med.*, **104**, 785.

LAUCIUS, J. F., BODURTHA, A. J., MASTRANGELO, M. J. & CREECH, R. H. (1974) Bacille Calmette-Guérin in the treatment of neoplastic disease. *J. Reticuloendothel. Soc.*, **16**, 347.

MCBRIDE, W. H., TUACH, S. & MARMION, B. P. (1975) The effect of gold salts on tumour immunity and its stimulation by *Corynebacterium parvum.* *Br. J. Cancer*, **32**, 558.

MÖLLER, G. (ed.) (1976) Experiments and the concept of immunological surveillance. *Transplant. Rev.*, **28**.

NATHAN, C. F., ASOFSKY, R. & TERRY, W. D. (1977) Characterization of the nonphagocytic adherent cell from the peritoneal cavity of normal and BCG-treated mice. *J. Immunol.*, **118**, 1612.

NELSON, D. S. (ed) (1976) *Immunobiology of the Macrophage.* New York: Academic Press.

OJO, E., HALLER, O., KIMURA, A. & WIGZELL, H. (1978) An analysis of conditions allowing *Corynebacterium parvum* to cause either augmentation or inhibition of natural killer cell activity against tumor cells in mice. *Int. J. Cancer*, **21**, 444.

PETO, R., ROE, F. J. C., LEE, P. N., LEVY, L. & CLACK. J. (1975) Cancer and ageing in mice and men. *Br. J. Cancer*, **32**, 411.

SCOTT, M. T. (1974) *Corynebacterium parvum* as an immunotherapeutic anti-cancer agent. *Semin. Oncol.*, **1**, 367.

STUTMAN, O. (1977) Immunodeficiency and cancer. In *Mechanisms of Tumor Immunity*. Eds. I. Green, S. Cohen, & R. T. McCluskey. New York: John Wiley. p. 27.

TELLER, M. N. (1972) Interrelationships among Aging, immunity and cancer. In *Tolerance, Autoimmunity and Aging*. Eds. M. M. Sigel & R. A. Good. Springfield: Thomas p. 18.

THOMSON, A. W. & FOWLER, E. F. (1977) Potentiation of tumour growth by carrageenan. *Transplantation*, **24**, 397.

ZINZAR, S. N., SVET-MOLDAVSKY, G. J. & KARMANOVA, N. V. (1976) Nonimmune and immune surveillance. I. Growth of tumors and normal fetal tissues grafted into newborn mice. *J. Natl Cancer Inst.*, **57**, 47.